# In-Person or Online? The Effect of Delivery Mode on Team-Based Learning of Clinical Reasoning in a Family Medicine Clerkship

**DOI:** 10.3390/medsci10030041

**Published:** 2022-08-08

**Authors:** Oksana Babenko, Mao Ding, Ann S. Lee

**Affiliations:** Department of Family Medicine, Faculty of Medicine & Dentistry, University of Alberta, Edmonton, AB T6G 2R3, Canada

**Keywords:** team-based learning, online learning, medical students, clinical reasoning, clinical decision making

## Abstract

In health professions education, team-based learning (TBL) has been used to help learners develop clinical reasoning and decision-making skills. The COVID-19 pandemic has challenged institutions to move curriculum delivery from largely in-person to online. With the anticipated return to in-person instruction and arguments made in favor of online instruction in certain circumstances, evidence is needed to support decision making in curriculum planning. The purpose of this study was to examine the effect of delivery mode (in-person vs. online) on student learning of clinical reasoning and clinical decision-making (CR/CDM) in the family medicine clerkship. Data from three cohorts of third-year medical students were included in the study: 2018/2019 cohort, in-person; 2019/2020 cohort, half of the cohort in-person, half of the cohort online; 2020/2021 cohort, online. Students’ performance data—individual readiness assurance test (IRAT) and group readiness assurance test (GRAT) scores—were used. The Generalized Estimating Equations (GEE) analysis was performed. As expected, students scored higher in GRAT than IRAT across the three cohorts. No significant IRAT-GRAT differences were observed between in-person and online delivery of TBL sessions. Student learning of CR/CDM in TBL is comparable between the two modes of delivery in the family medicine clerkship. Future research in other clerkships, years of medical education, and professional programs is needed to inform decision making regarding the TBL delivery mode.

## 1. Introduction

With peer collaboration at its core, team-based learning (TBL) is a small-group method of instruction designed to facilitate knowledge application and development of problem solving [1,2,3]. Traditional TBL comprises three main components: preparation, readiness assurance, and application [4]. In preparing for the TBL session, students are asked to review reading materials and familiarize themselves with key concepts and learning objectives. For readiness assurance, students first take an individual readiness assurance test (IRAT), which serves as a measure of students’ understanding of the material and their independent problem solving. After the IRAT, students are assigned to small groups and take the same test (i.e., group RAT or GRAT), collaboratively answering questions through consensus-building discussion. After finalizing answers in small groups, students receive immediate feedback from the instructor. In a meta-analysis of TBL studies, Ngoc and colleagues reported that students achieved significantly higher scores on the GRAT compared to the IRAT [2,5,6,7,8,9,10,11,12,13,14,15]. The authors concluded that “the improvement from the IRAT to the GRAT could be regarded as the learning and progress occurring in the Zone of Proximal Development (ZPD), as the result of students collaborating with one another” [2,16]. Finally, in the application component, students are presented with practical problems and are asked to apply their knowledge to solve the problems or propose possible solutions [4].

In the education of health professionals specifically, TBL has been extensively used to help learners develop reasoning and decision-making skills, key skills for successful clinical practice [2,3,17]. The benefits of TBL have been well documented, including enhanced collaboration, performance, and clinical competencies in health professions trainees [1,2,18,19].

Recently the COVID-19 pandemic has challenged learning and instruction profoundly. Curriculum delivery, including in medical programs, had to shift swiftly from largely in-person to online. While online learning appears to support collaboration and communication, it can be challenging, posing difficulties in rapport building and limiting the depth of discussion [20]. Furthermore, existing literature remains sparse and inconclusive about the effect of delivery mode (in-person vs. online) on student learning in small-group methods of instruction, including in TBL [21,22,23,24,25].

Specifically, River and colleagues’ systematic review of technology-blended TBL studies [26,27,28,29,30,31,32,33,34] in health professions education reported varying degrees of effects on exam performance and learners’ perceptions [21]. Palsolé and Awalt reported no significant differences in grades following online TBL in undergraduate science students compared to in-person TBL; however, students reported greater satisfaction with teamwork in online TBL compared to in-person [22]. Gruenberg and colleagues also reported no significant differences in pharmacy students’ therapeutic reasoning exam performance between in-person and online small-group learning; however, the majority of students were in favor of the online format [24]. Holmes observed no significant differences in IRAT scores between in-person and online TBL in a cohort of 75 graduate physician assistant students; however, significant differences were present in GRAT scores, with a higher mean score in the in-person TBL format [25]. DeMasi and colleagues found a difference in student perception, but not in performance in a course with 55 immunology students [23]. Silva and colleagues observed no significant differences in undergraduate students’ preferences regarding the TBL sessions being in-person vs. online [3]. Interestingly, prior to the pandemic, learners tended to favor the in-person over the online format [21,23], whereas studies published shortly after the start of the pandemic report learners favoring the online format equally or even more so than the in-person format [3,24].

The reality of the on-going pandemic, combined with an interest in continued online instruction in certain circumstances after the pandemic, has created an urgency for evidence of whether online delivery enhances or hinders student learning. With the anticipated return to in-person instruction, as well as arguments made in favor of online instruction due to the flexibility it affords in terms of location, timing, and facilitator recruitment [19], evidence is needed to support decision making. In TBL specifically, where rapport building and collaborative discussion are key, curriculum planners want to know if online delivery hampers collaborative learning of students in TBL, and if so, this method may be better suited for in-person instruction.

As such, the purpose of the present study, drawing on three years of data before and during the pandemic, was to examine whether the delivery mode affects collaborative learning of students in TBL. To this end, we compared medical students’ scores in academic sessions—when delivered in-person vs. online—in the family medicine core clerkship, in which TBL is used to help students develop clinical reasoning and clinical decision making (CR/CDM). Specifically, we compared the change from IRAT to GRAT scores between the two delivery modes to determine if there are differences in collaborative learning of CR/CDM in TBL. This would provide much needed evidence for educators to make decisions around delivery of TBL.

## 2. Materials and Methods

### 2.1. Participants

Participants in this study were third-year medical students at the University of Alberta, Canada. The third and fourth years in the medical program are clinical years, the time when students actively develop CR/CDM skills. In the third year specifically, TBL is a method of instruction implemented in several core clerkships to facilitate CR/CDM skills development by having students engage in consensus-building discussion as they work on case-based scenarios in small groups. The entire instructional activity takes place in the following sequence: prior to the session, students are asked to review the learning objectives and key concepts; at the beginning of the session, students first work individually on clinical case-based scenarios and answer a set of questions (i.e., IRAT) as they pertain to each scenario; after the IRAT, students are randomly assigned to groups of 3–4 students and work collaboratively on the scenarios and together answer the same questions by engaging in consensus-building discussion (i.e., GRAT). Two clinician teachers score the answers and facilitate discussion to provide feedback to students on their performance on the clinical scenarios. The use of clinical case-based scenarios, purposefully designed to facilitate CR/CDM learning, aims to fulfill the application component of the TBL method. The clerkship director, who oversees the CR/CDM sessions in family medicine, including the development of clinical case-based scenarios, ensures the difficulty of the scenarios is comparable across the years. The difficulty of the scenarios as well as the targeting and clarity of the questions and distractors are peer-reviewed by clinician teachers in family medicine.

This method of instruction is currently implemented in the core clerkships in pediatrics (session A) and family medicine (session B). All third-year medical students participate in both sessions, the order of which (AB or BA) is determined by the overall clerkship schedule. Due to the data availability in this study, only the scores from the family medicine clerkship were used in the analyses. However, because we anticipated some learning with this method of instruction to take place with its repeated use, we hypothesized that students who had a session in a preceding clerkship would have improved scores in the subsequent session. As such, in the analyses of family medicine scores, we controlled for the session order (i.e., whether the family medicine session was before or after the pediatrics session). An institutional ethics approval was obtained prior to data analyses.

### 2.2. Data

We used IRAT and GRAT scores (both range from 0 to 100) from three cohorts of third-year medical students, specifically the 2018/2019 cohort (145 students, 43 small groups), the 2019/2020 cohort (148 students, 43 small groups), and the 2020/2021 cohort (146 students, 40 small groups). The 2018/2019 cohort experienced the CR/CDM sessions solely in-person. Half of the 2019/2020 cohort experienced the CR/CDM sessions in-person and the other half of the cohort experienced CR/CDM sessions online when the COVID-19 pandemic forced instruction to be shifted online using Zoom for group work and facilitate discussion in March 2020; this resulted in 22 small groups in-person and 21 small groups online in this cohort. The 2020/2021 cohort experienced the CR/CDM sessions solely online. In total, there were 439 students in 126 small groups, with 61 (48%) groups experiencing TBL online and 65 (52%) groups experiencing TBL in-person.

### 2.3. Analyses

All analyses were performed in SPSS 25.0 (IBM Corp., Armonk, NY, USA). Given the clustered nature of the data (i.e., students nested within respective cohorts), we used the Generalized Estimating Equations (GEE) [35] to account for correlations present in the clustered data. The GEE combines the features of analysis of variance and regression analysis. Specifically, it allows for significance testing of group means (as in analysis of variance) and individual predictors (as in regression analysis). As a semi-parametric technique, however, the GEE does not require satisfaction of the assumptions of normality, homogeneity of variances, and homoscedasticity, which are required in analysis of variance and regression analysis.

In the GEE analysis in this study, the dependent variable (outcome) was the difference in GRAT scores and the average of IRAT scores of students in corresponding small groups. The independent variables (predictors) were cohort (2018/2019, 2019/2020, 2020/2021), delivery mode (in-person vs. online), and session order (the family medicine session took place before or after the pediatrics session). Significance level was set to 0.05; Bonferroni correction was applied in the case of multiple comparisons of group means.

## 3. Results

For the in-person delivery mode, the observed mean IRAT and GRAT scores were 61.39 (SD = 7.20) and 68.85 (SD = 8.93), respectively. For the online delivery mode, the observed mean IRAT and GRAT scores were 54.81 (SD = 8.93) and 66.28 (SD = 9.85), respectively.

The results of the GEE analyses are provided in Table 1. Specifically, Table 1 shows estimated mean differences between the IRAT and GRAT scores if each cohort were to experience TBL sessions in-person vs. online. Although the estimated score differences between the IRAT and the GRAT (the far-right column in Table 1) appear to be consistently higher in the online delivery than in the in-person delivery (the middle column in Table 1), these differences within each cohort were statistically non-significant. When comparing the 2018/2019 cohort (pre-pandemic) and 2020/2021 cohort (following the acute pandemic phase) only, the respective mean differences were also non-significant.

Across the three cohorts, the estimated mean difference between students’ IRAT and GRAT scores was 9.30 (standard error (SE) = 0.68; 95% CI: 7.98–10.63). The GEE analysis also indicated that the three predictors considered in the study were not significant in explaining the variability in the dependent variable (i.e., IRAT and GRAT score differences): cohort (Wald χ^2^ = 0.561, df = 2, *p* = 0.756), delivery mode (Wald χ^2^ = 2.136, df = 1, *p* = 0.144), and session order (Wald χ^2^ = 0.386, df = 1, *p* = 0.534).

## 4. Discussion

In the present study, based on the GEE analysis of three years of TBL performance data (i.e., students’ IRAT and GRAT scores) collected before and during the pandemic, we found no significant differences in estimated mean scores between the in-person and online modes of TBL delivery. Further, the mean differences in scores between the IRAT and the GRAT in the current study were comparable to the mean differences reported in the meta-analysis of 11 studies in which TBL delivery was in-person [2]. This finding provides evidence that CR/CDM learning in TBL is comparable between the two modes of delivery, as evident in score differences being of similar size in the in-person and online TBL sessions.

This finding has important implications for practice. As the pandemic becomes endemic, our results help balance risks and benefits, and inform decisions around what methods of instruction need to be in person and what can, in fact, be delivered online, while ensuring student learning is not negatively affected by the choice of delivery mode. Online delivery is a safer option for health as it can limit the spread of illness when students are in clinical rotations and have many patient interactions. Furthermore, the online format offers flexibility in delivering content and recruiting session facilitators, who are often busy practicing clinicians. Finally, it is also a desirable option for distributed programs to deliver instruction to trainees in distributed or remote sites, reducing travel time and costs as well as carbon footprint. However, these benefits cannot be at the expense of student learning.

The COVID-19 pandemic forced institutions to try alternative modes of instruction delivery, resulting in unexpected benefits and challenging existing assumptions. Before the pandemic the online delivery of TBL and similar small-group methods of instruction (e.g., problem-based learning) were rarely considered in the literature due to well-recognized limitations of collaborative work online and challenges replicating in-person interaction and dynamics of active learning [3,20]. Our results suggest learning in TBL similarly occurs in online and in-person formats despite these limitations.

## 5. Limitations and Strengths

Several important limitations need to be considered. First, the data for this study came from third-year medical students in one institution. We aimed to counterbalance this limitation by analyzing the data from three cohorts of third-year medical students. Nonetheless, due to data availability, the sample size in this study was convenient rather than sufficiently powered to be able to detect a significant difference between the two modes of delivery.

Furthermore, due to the retrospective nature of the study (i.e., using the data that was collected before and during the pandemic), delivery mode is confounded with the pandemic period. That is, pre-pandemic the CR/CDM sessions were delivered in-person whereas during the pandemic the sessions were forced to be exclusively delivered online due to health and safety concerns. Nevertheless, the mean improvement between IRAT and GRAT in the sessions delivered in-person before the pandemic and online during the pandemic were not significantly different. The trend toward the larger mean improvement between IRAT and GRAT in case of the sessions delivered online during the pandemic needs to be considered in light of the lower mean score on IRAT. That is, on average these students had more room to improve than their counterparts pre-pandemic who on average had higher IRAT scores.

Next, we had access only to the assessment data (students’ scores) in the family medicine clerkship. We accounted for this limitation by including the session order (i.e., family medicine clerkship took place before or after the pediatrics clerkship) as a variable in the analysis. Neither were we able to assess the association of in-person vs. online TBL on the overall clerkship performance. However, we did not have missing data because we used assessment data (as opposed to self-report data) available for all the students in each cohort and as such, response bias was not an issue.

Using the GEE analysis, we were able to account for the fact that the data used in this study was clustered or nested data (i.e., students were nested within respective cohorts). As a semi-parametric technique, this type of analysis also helped increase statistical power to detect significant effects if they were truly present in the data.

Finally, this study focused on quantitative data to examine the effect of TBL delivery on collaborative learning. Future studies to understand whether mode of delivery impacts the development of other skills for clinical practice such as teamwork may provide additional outcomes for decision making.

## 6. Conclusions

The results of this study suggest that TBL supports students to attain a similar level of collaborative learning in a family medicine clerkship irrespective of delivery mode. In making delivery decisions, educators need to consider the weighing of the risks, benefits, and limitations of in-person and online delivery, including local context, with the literature providing mixed insights into the changing perceptions of and preferences for online learning since the beginning of the COVID-19 pandemic. Further research in other clerkships, professional programs, and other years of studies, as well as additional outcomes (e.g., student preferences and perceptions) is needed to aid decision making now and beyond the pandemic.

## Figures and Tables

**Table 1:** Estimated mean differences (standard errors) between IRAT and GRAT scores based on delivery mode (with cohort, delivery mode, and session order entered as predictors in the GEE analysis).

Cohort	# Students; # Small Groups	Delivery Mode	
		In-Person EM (SE)	Online EM (SE)
2018/2019	145 students; 43 groups	7.24 (1.05)	10.10 (2.20)
2019/2020	148 students; 43 groups	7.56 (1.41)	10.42 (1.40)
2020/2021	146 students; 40 groups	8.82 (2.26)	11.68 (1.09)
overall	439 students; 126 groups	7.87 (1.21)	10.73 (1.17)

#—number; EM—estimated mean; SE—standard error for the estimated mean.

## Data Availability

The data analyzed in this study are available on request from the corresponding author. The data are not publicly available due to privacy/ethical considerations.
